# Vitamin D Levels in COVID-19 and NonCOVID-19 Pediatric Patients and Its Relationship with Clinical and Laboratory Characteristics

**DOI:** 10.3390/biomedicines12040905

**Published:** 2024-04-18

**Authors:** Maria Totan, Ioana-Octavia Matacuta-Bogdan, Adrian Hasegan, Ionela Maniu

**Affiliations:** 1 Faculty of Medicine, Lucian Blaga University of Sibiu, 2A Lucian Blaga Str., 550169 Sibiu, Romania; maria.totan@ulbsibiu.ro (M.T.); adrian.hasegan@ulbsibiu.ro (A.H.); 2 Clinical Laboratory, Pediatric Clinical Hospital Sibiu, 2-4 Pompeiu Onofreiu Str., 550166 Sibiu, Romania; 3 Research Team, Pediatric Clinical Hospital Sibiu, 550166 Sibiu, Romania; 4 Research Center in Informatics and Information Technology, Mathematics and Informatics Department, Faculty of Sciences, Lucian Blaga University of Sibiu, 550025 Sibiu, Romania

**Keywords:** vitamin D, COVID-19, SARS-CoV-2, children, pediatric, laboratory markers, symptoms

## Abstract

25-hydroxyvitamin D [25(OH)D] is a marker with an important role in regulating the inflammatory response. Low concentrations of this vitamin are often found among the population, correlated with increased risk of respiratory tract infections. The aim of the study is to evaluate the relationship between vitamin D levels and clinical and laboratory markers in children and adolescents hospitalized with and without COVID-19. A retrospective study, including all patients tested for SARS-CoV-2 and having vitamin D measured, was performed. All included hospitalized cases, 78 COVID-19 patients and 162 NonCOVID-19 patients, were divided into subgroups according to their 25(OH)D serum levels (<20 ng/mL—deficiency, 20–30 ng/mL—insufficiency, ≥30 ng/mL—normal or <30 ng/mL, ≥30 ng/mL) and age (≤2 years, >2 years). Vitamin D deficiency and insufficiency increased with age, in both COVID-19 and NonCOVID-19 groups. All symptoms were encountered more frequently in cases of pediatric patients with COVID-19 in comparison with NonCOVID-19 cases. The most frequently encountered symptoms in the COVID-19 group were fever, loss of appetite, and nasal congestion. In the NonCOVID-19 group, serum 25(OH)D concentrations were positively correlated with leukocytes, lymphocytes, and LMR and negatively correlated with neutrophils, NLR, and PLR while no significant correlation was observed in the case of COVID-19 group. Differences between vitamin D status and clinical and laboratory parameters were observed, but their clinical significance should be interpreted with caution. The results of this study may offer further support for future studies exploring the mechanisms of the relationship between vitamin D and clinical and laboratory markers as well as for studies investigating the implications of vitamin D deficiency/supplementation on overall health/clinical outcomes of patients with/without COVID-19.

## 1. Introduction

Vitamin D (sunshine vitamin) is synthesized from 7-dehydrocholesterol at the dermis level (under the action of UVB light of 290–315 nm wavelength) or from the diet/supplements [1]. To obtain a biologically active form, vitamin D3 requires two hydroxylation steps (first in the liver under the action of 24,25-hydroxylase enzyme, second in the kidneys under the action of renal 1-α-hydroxylase enzyme). The resulting active forms 1.25(OH)2 vitamin D which has limited clinical significance and a short half-life in circulation (approximately 4–8 h). Of the two resulting forms, 25(OH)D and 1.25(OH)2D, the first reflects more accurately the vitamin D status because it is more easily measurable (concentration pmol vs. nmol levels), and its half-life circulation (2–3 weeks vs. 4–8 h) [2], 1.25(OH)2D, could be influenced by PTH (parathyroid hormone) [2]. Vitamin D has a role in calcium and phosphorus homeostasis, bone metabolism, and immune response modulation (through several mechanisms) [3]. The implications of vitamin D in immunity has been analyzed more and more in recent years. The presence of vitamin D receptors in a vast majority of immune cells suggests that vitamin D has regulatory functions of both the innate and adaptive immune system [4]. Many studies showed that supplementation of vitamin D exerts a protective role against respiratory infections [5,6]. Previous studies analyzed the relationship/role of vitamin D levels/supplementation in incidence/severity of respiratory tract infections, including COVID-19 [7,8,9]. Studies regarding the relationship between vitamin D and COVID-19 clinical and laboratory profile or disease outcomes, mainly focused on adult patients and often showed conflicting results [10,11,12,13,14,15,16,17,18,19,20,21,22]. A series of studies identified a negative correlation between vitamin D and NLR and CRP [12,13], while other studies did not identify any correlation between them [14,15,16,17]. The study of Yarali et al. [18] reported that in SARS-CoV-2 infection in children, the leukocytes count was normal, while the study of Yamada et al. [19] reported the association between leukocytosis and a poor outcome. The magnesium levels were reported as low and correlated with disease severity by studies [20,21], while the study [22] showed that hypomagnesemia is related with moderate cases. This study aimed to describe and evaluate the vitamin D levels and its association with clinical and laboratory markers in children hospitalized with and without COVID-19 from our geographic area.

## 2. Materials and Methods

We retrospectively evaluated the electronic medical records for patients (aged under 18 years) who were tested for SARS-CoV-2 by nasopharyngeal swab RT-PCR analysis and had measured serum 25(OH)D concentration at admission for hospitalization in any ward of the Pediatric Clinical Hospital of Sibiu in the period from July 2022 to May 2023. Informed consent was obtained from all patients’ guardians for all subjects included in this study in accordance with the Declaration of Helsinki. The study was approved by the Institutional Review Board of Pediatric Clinical Hospital of Sibiu (No. 2218). Retrospective data including demographic, clinical, and laboratory results were analyzed. 

Laboratory examinations included serum biomarkers (calcium (mmol/L), magnesium (mmol/L), urea (mg/dL), creatinine (mg/dL), iron (µmol/L), AST—asparate aminotransferase (U/L), ALT—alanine aminotransferase (U/L), CRP—C-reactive protein (mg/L); hematological parameters (hemoglobin (g/dL), leukocyte (×10^3^/µL), neutrophil (%), lymphocytes (%), monocytes (%), basophils (%), platelet (×10^3^/µL), NLR—neutrophil lymphocyte ratio, PLR—platelet lymphocytes ratio, LMR—lymphocytes monocytes ratio, RBC—erythrocite (×10^6^/µL), MCV—erythrocite mean cell volume (fL), MCH—mean cell hemoglobin (pg), MCHC—mean cell hemoglobin concentration (g/dL)), and immunological tests (25(OH)D (ng/mL)). We considered normal range references, adjusted to age and gender, according to the laboratory guidelines where the study took place [23].

Serum 25(OH)D concentration was measured by chemiluminescence immunoassay method using a Biomerieux kit (Craponne, France). Vitamin D deficiency, insufficiency, and normal values were defined as 25(OH)D levels: <20 ng/mL—deficiency, 20–30 ng/mL—insufficiency, >30 ng/mL—normal [24]. Serum biomarkers were measured using an automated biochemical analyzer, Abbott, C 4000 (Abbott Park, IL, USA). Hematological parameters were measured using an automated hematological analyzer Sysmex XS-1000i (Burladingen, Germany). A confirmed case of COVID-19 was defined as a positive result real-time reverse transcriptase-polymerase-chain reaction (RT-PCR) assay of nasal and pharyngeal swab specimen, using automatic extraction, on a 24-position extractor Lab-Aid 824-Zeesan, and the amplification is done on a BIORAD CFX2s device (Hercules, CA, USA).

Data were analyzed using descriptive statistics. Categorical variables were expressed as counts and percentages and continuous variables were expressed as medians and interquartile range (IQR: 25th percentile–75th percentile). The Shapiro–Wilk test was used to assess the normal distribution of continuous variables. Comparisons across different groups were conducted using the Mann–Whitney U test or Kruskal–Wallis test to determine differences in medians and the Chi-Square test or Fischer exact test to determine differences in proportions. Correlations between vitamin D levels and laboratory variables were analyzed using Spearman’s rank correlation coefficient. Statistical analyses were performed using SPSS v.20 and R v.4.0.5 software.

## 3. Results

A total of 240 patients tested for SARS-CoV-2 and having vitamin D measured were included in this study. Of this, 78 were hospitalized for COVID-19 and 162 were NonCOVID-19 patients, hospitalized for other pathologies. Regarding gender, the COVID-19 group had 52.56% males (*n* = 41), and a similar percentage was encountered in the NonCOVID-19 group (56.79%, *n* = 92). The median age in the COVID-19 group was 10 months while in the NonCOVID-19 group the median age was higher (90.50 months). We divided each group of patients into three subgroups according to their 25 (OH) D serum levels. The number of cases with vitamin D deficiency and insufficiency increased with age, in both COVID-19 and NonCOVID-19 groups, and the percentages were higher in COVID-19 patients compared to NonCOVID-19 patients (COVID-19 vs. NonCOVID-19: <1 year—15.55% vs. 8.70%; 1–2 years—20.00 % vs. 7.69%; 2–6 years—33.34% vs. 15.62%, >6 years—87.5% vs. 63.8%). There were no statistical differences between gender and vitamin D levels. All symptoms were encountered more frequently in cases of pediatric patients with COVID-19 in comparison with NonCOVID-19 cases (*p* < 0.000). The most frequently encountered symptoms in the COVID-19 group were fever (87.18%), loss of appetite (57.69%), and nasal congestion (50%). Comparison of demographic features and clinical symptoms of patients between children with and without COVID-19 who had deficient, insufficient and normal levels of vitamin D are shown in Table 1. 

We analyzed the correlation between vitamin D and various inflammatory and hematological parameters. We divided each group of patients into two subgroups according to their age (age ≤ 2 years, age > 2 years) and the results are presented in Table 2. We also performed a comparative analysis of median values of laboratory characteristics according to vitamin D status and age and the results are presented in Table 3. 

It can be observed that, in the case of both age groups, calcium levels and serum magnesium concentration were lower for children with COVID-19 infection and vitamin D < 30 ng/mL compared to the other subgroups. Creatinine levels in children over 2 years old are higher in patients with COVID-19 and vitamin D < 30 ng/mL (median: 0.60, IQR: 0.50–0.72) compared to patients with COVID-19 and vitamin D ≥ 30 ng/mL (median: 0.48, IQR: 0.45–0.55). This trend is also observed in NonCOVID-19 patients (over 2 years old). Regardless of age, iron levels are lower in COVID-19 compared to NonCOVID-19 patients. In children under 2 years old, iron was lower in patients with COVID-19 and vitamin D < 30 ng/mL compared to other subgroups. In both age groups, in the case of COVID-19 patients, a positive association was observed between neutrophils and vitamin D (age ≤ 2 years: 37.10 vs. 41.10; age > 2 years: 64.60 vs. 69.20), and in the case of NonCOVID-19 patients a negative association was observed between neutrophils and vitamin D (age ≤ 2 years: 18.60 vs. 16.70; age > 2 years: 48.80 vs. 40.90). In the NonCOVID-19 group, serum 25(OH)D concentrations were positively correlated with leukocytes (r = 0.203, *p* = 0.010), lymphocytes (r = 0.428, *p* = 0.000), and LMR (r = 0.332, *p* = 0.000) and negatively correlated with neutrophils (r = −0.435, *p* = 0.000), NLR (r = −0.439, *p* = 0.000), and PLR (r = −0.267, *p* = 0.001), while no significant correlation was observed in the case of the COVID-19 group. In both COVID-19 and NonCOVID-19 groups, positive correlations were encountered with AST (COVID-19: r = 0.247, *p* = 0.041; NonCOVID-19: r = 0.466, *p* = 0.000) and magnesium (COVID-19: r = 0.470, *p* = 0.036; NonCOVID-19: r = 0.350, *p* = 0.000), while negative correlations were encountered with MCV (COVID-19: r = −0.330, *p* = 0.003; NonCOVID-19: r = −0.355, *p* = 0.000) and MCH (COVID-19: r = −0.270, *p* = 0.017; NonCOVID-19: r = −0.183, *p* = 0.020). 

## 4. Discussion

Recent studies on vitamin D investigated its association in multiple metabolic, physiological, and immunological processes. Its effects on both innate and acquired immunity, cellular and humoral, have been extensively analyzed [8,25,26,27]. Our study evaluated both clinical and laboratory parameters in correlation with the level of vitamin D in children hospitalized with COVID-19 compared to those without this infectious pathology. 

The most frequently encountered symptoms in the COVID-19 group were fever, loss of appetite, and respiratory symptoms. Fever was more common in COVID-19 patients (87.18% vs. 8.33%) with normal vitamin D levels in both studied groups (73.52% vs. 63.63%). The next most frequent symptom in the COVID-19 group was decreased appetite followed by respiratory symptoms, including cough (COVID-19: 83.78% vs. Non COVID-19: 71.42%), rhinorrhea (COVID-19: 80% vs. Non COVID-19:57.14%), nasal congestion (COVID-19: 71.79% vs. Non COVID-19: 70%), and breathing difficulties occurring only in COVID-19-positive patients. Fever as a symptom of SARS-CoV-2 infection was reported with varying frequency in pediatric patients, ranging from 43.5% to 90.9% [7,14,28,29,30,31,32,33,34]. The results obtained are similar to those reported by Alpcan et al. [14], who identified fever as the most frequent symptom (61.3%) in patients infected with SARS-CoV-2. They also found that patients with low levels of vitamin D had a lower incidence of fever. Moreover, they note that among respiratory symptoms, cough is the most frequent, followed by dyspnea and rhinorrhea. Heidari et al. reported a higher rate in patients with normal vitamin D levels [35]. In their study, Yilmaz and colleagues [7] identified fever as a symptom in one-third of COVID-19 patients and also a negative correlation between fever and vitamin D, a conclusion similar to that of the study conducted by Shah et al. [36]. Fever correlates with inflammation and cytokines released during SARS-CoV-2 infection. A special role is played by prostaglandin E, which modulates fever but is also correlated with cough. Headache occurs in nearly a quarter of children infected with SARS-CoV-2 included in the study, and in half of the cases, it occurs in subjects with normal serum levels of vitamin D. Alpcan et al. reported a frequency of 12% for this symptom [14].

Hematological parameters are affected in the context of infections, including SARS-CoV-2 infection. Our study showed that, in the case of children under 2 years old, hemoglobin levels were lower in patients with vitamin D < 30 ng/mL, compared to those with vitamin D ≥ 30 ng/mL, in both groups. In our population, iron-deficient anemia is the most common nutritional disease in children under 2 years of age due to the lack of iron supplementation and nutritional habits. Studies showed controversial results. Some reported that lower hemoglobin levels are associated with the severity of COVID-19, especially multiple system inflammatory syndrome [37], while other studies showed no abnormalities in red blood cell count or level of hemoglobin even in severe disease [38,39,40,41]. 

The current study shows that leukocyte and lymphocyte levels (regardless of age) are lower in COVID-19 patients compared to NonCOVID-19 patients. Serum 25(OH)D concentrations were positively correlated with leukocytes and lymphocytes in the NonCOVID-19 group. Studies [42,43] reported a positive correlation between serum concentrations of vitamin D and lymphocyte count. This finding is consistent with other studies that show that low leukocyte count is one of the hematological modified parameters. The reported percentages vary in wide ranges, from 19% to 47% for different studies [44,45,46]. In the study conducted by Yarali et al., it was shown that most children with COVID-19 had a normal leukocyte count [18]. In the meta-analysis of Yamada et al. on 18 studies that included 3278 patients, leukocytosis was associated with poor outcomes while leukopenia was associated with a better prognosis [19]. Other authors concluded that, for children, leukocyte count may not be a reliable laboratory marker to assess the severity of the disease [46]. Many mechanisms are responsible for lymphopenia found in COVID-19. SARS-CoV-2 virus binds to ACE-2 receptors on the surface of the lymphocytes, invades them and causes them to decompose. The cytokines released during inflammation may induce lymphocyte apoptosis and a disruption in their turnover, contributing to the low lymphocyte count [42,47]. Compared to adults with COVID-19, lymphopenia is rare in children and correlated to more severe forms of the disease. Children naturally have more natural killer cells and therefore lymphopenia is not so frequent [37,38,41,46,48,49].

In terms of neutrophils, we encountered elevated levels in COVID-19 compared to NonCOVID-19 patients, regardless of age. Moreover, in the COVID-19 group, there is a positive association between neutrophils and vitamin D, and for the NonCOVID-19 group we found a negative association between neutrophils and vitamin D in both age groups. The results of the various studies we refer to are contradictory. Some of them report neutropenia to be more prevalent compared to neutrophilia. In the study of Yarali et al. [18], neutropenia was noted in 23.3%, compared to neutrophilia (13.3%), and over 14% from the children with COVID-19 included in the study of Guner Ozenen et al. [50] had neutropenia. Similar results were reported by Argun et al. [45], but his study only included 33 children. In studies [12,18,44,45,50], neutrophilia was associated with the severity and the prognosis of the SARS-CoV-2 infection, especially in those children who developed multi-system inflammatory syndrome (MIS-C). In his study on 26 patients, Pimentel et al. found a higher neutrophil count in the low vitamin D group, but no correlations between vitamin D concentration in the serum and neutrophil count were identified [51].

Our study revealed that, regardless of age, the NLR ratio is higher in COVID-19 when compared to NonCOVID-19 patients. In COVID-19, the neutrophil to lymphocyte ratio was associated with the extent of symptoms, disease severity, and poor outcome for both adults and children [13,37,52,53,54,55,56,57]. The association between NLR and vitamin D is not yet fully understood. The study of Renieris et al. [15] and Pimentel et al. [51] showed that NLR was inversely associated to vitamin D levels, while Gulcan et al. [58] did not identify any correlation between the two parameters. 

The study that we conducted showed that thrombocytes tend to be lower for all COVID-19 patients, with the lowest value in children over 2 years old and with vitamin D < 30 (median 240.50, IQR: 220.00–316.50 × 10^3^/μL); 2.6% of COVID-19 patients had thrombocytopenia (<150 × 10^3^/μL). We also noted that in COVID-19 children over 2 years of age, there is a positive trend between platelets and vitamin D. Beyond its classic role in calcium metabolism, vitamin D has other functional roles. The vitamin D deficiency has a critical role in coagulation, inflammation, thrombosis, and endothelial dysfunction and it has been implicated in immunological diseases. In the study of Salamanna et al. [59], the explanation proposed was an increased pro-inflammatory cytokine release and via oxidative stress stimulated megakaryopoiesis and PLT activation. The decrease in platelet count is not common in patients with COVID-19. Studies reported variable percentages, going from 4.8% up to 53.6% [37,60,61,62,63]. These studies proposed several mechanisms for thrombocytopenia found in COVID-19, such as a reduction in thrombopoiesis due to direct infection of bone marrow, SARS-CoV-2 inhibition of bone marrow hematopoiesis, destruction of bone marrow progenitor cells by cytokine storm, excessive destruction of the platelets in the immune processes, and platelet aggregation in the lungs. In their meta-analysis, Lippi et al. [64] showed that a low platelet count was related to a threefold enhanced risk of severe illness and it was a significant factor in mortality of COVID-19 patients. The study [35] reported that in children with COVID-19, the platelet count was lower in many cases compared to adult patients and it was closely related to the severity of the disease. 

In the conducted study, a series of biochemical parameters were monitored. We found that the CRP values are higher in the group with COVID-19 compared to the NonCOVID-19 group, regardless of the age of the subjects. The results reported by the different studies are contradictory. A similar conclusion as the one drawn from our study is supported by other studies. These show that CRP values are correlated with the severity of the disease and more elevated in the early stages of inflammatory response [12,28,65,66,67,68,69]. Most studies have identified an inverse association between CRP and vitamin D levels [43,50,69,70,71,72,73]. On the other hand, the studies of Alpcan et al. [14] and Pizzini et al. [74] showed no correlation between vitamin D and CRP. The relationship between CRP and vitamin D in COVID-19 is complex. C-reactive protein is a pentameric protein synthesized by the liver as a response to inflammation of various causes. Its secretion is mainly induced by IL-6. The inflammatory cells involved in inflammation express the nuclear receptor for vitamin D, lowering its serum levels. This leads to the conclusion of an inverse association between CRP and vitamin D concentration. Low levels of vitamin D itself induce cytokines such as TNF-α and IL-1p responsible for low-grade inflammation and high levels of CRP [70]. On the other hand, apparently in COVID-19 patients, high levels of CRP are responsible for decreasing vitamin D levels, making it a “negative acute phase reactant” [71]. 

The study conducted has revealed that serum levels of calcium, magnesium, and iron are lower in subjects with COVID-19 and vitamin D levels below the threshold value of 30 ng/mL. In both the COVID-19 and NonCOVID-19 groups, positive correlations were encountered between serum 25(OH)D and magnesium. One of the main roles of vitamin D is its involvement in phospho-calcic metabolism by stimulating the intestinal absorption and kidney reabsorption of calcium. Magnesium interacts with vitamin D metabolism by converting the inactive form of vitamin D to the active form. The enzymes involved in the activation require magnesium as a cofactor [75,76]. Recent studies showed magnesium as implicated in immune responses, regulating NK cells and CD8 killer T cells’ cytotoxicity, and decreasing monocyte inflammatory cytokine production. Magnesium deficiency is associated with depressed immune responses, macrophage inflammatory responses, chronic low-grade inflammation, increased inflammatory responses during viral infections, and an increased risk for an inflammatory cytokine storm. The studies [77,78] demonstrated that the prevalence of hypomagnesemia was higher in patients with COVID-19 compared to healthy individuals. In SARS-CoV-2 infection, serum magnesium level conditions the clinical outcome [79,80,81,82], severity of the disease, disease progression, and mortality [77,78]. Results of different studies are controversial. Some studies showed that higher levels of magnesium can exert a protective role in COVID-19 [22,82]. The study of Mardani et al. on patients admitted to Shahid Modarres Hospital in Tehran, Iran, demonstrated that magnesium levels at admission correlated with the risk of in-hospital death for COVID-19 patients [57]. Another retrospective study on hospitalized patients in Wuhan showed that hypomagnesemia was more prevalent in the critical group and non-survivors [20]. In their study, Quilliot et al. performed an analysis in a cohort of COVID-19 adult patients. They demonstrated that in moderate cases, the prevalence of hypomagnesemia was higher and the magnesium levels were significantly lower [22].

In our study, calcemia was lower in subjects with COVID-19 and vitamin D levels below 30 ng/mL. Alteration of the intestinal absorption can explain the changes in the serum calcium concentration, but there could be other mechanisms such as modifications in regulatory mechanisms through PTH and vitamin D, or even a direct action of the virus. In the course of viral infections, calcium ions are essential elements for viral replication, entry, virion maturation, and release [83,84,85]. Studies showed that the levels of total and ionized calcium decreased in COVID-19 patients [83]. Bara El-Kurdi et al. [86] showed that maintaining normal serum calcium levels may prevent severe illness. Calcemia appears associated with the severity and prognosis of COVID-19 infection [87,88]. The disruption of iron homeostasis has been described in COVID-19. The parameters correlated with iron showed deviations from normal values in patients infected with SARS-CoV-2, whether hospitalized or not. The greatest deviations were recorded in critically ill patients [89,90,91,92,93,94]. 

In the study that we conducted, serum creatinine was higher in infected patients with vitamin D levels above the threshold value. Children’s normal values for creatinine are highly age-dependent and influenced by sex, especially in adolescence when the muscle mass grows rapidly for males [95,96]. In a cohort study on over 1200 participants in Spain, González-Molero et al. demonstrated that 25-hydroxyvitamin D levels were correlated with creatinine in the normal population [97].

Our study has some limitations. It is a single-center, retrospective study, including a small sample size of patients with available 25(OH)D levels, considering unbalanced groups of patients (with different median ages) with COVID-19 but also NonCOVID-19 patients (with other pathologies that could determine variation in serological parameters). Also, the clinical significance of the results should be interpreted with caution due to the fact that the clinical evaluation of the patients and the determination of the biological parameters were carried out at different stages of the disease and were not assessed dynamically; due to the thresholds used for comparison (for levels of 25(OH)D, for age); due to inclusion of a relatively narrow number of (available/reported) symptoms and laboratory markers, and to any other potentially induced bias.

## 5. Conclusions

The analysis of the data, using continuous or categorical values of 25(OH)D, in the COVID-19/NonCOVID-19 groups and subgroups, provide insights regarding the relationship between vitamin D and clinical and laboratory markers. The results of this study may offer further support for future studies exploring the mechanisms of the relationship between vitamin D and clinical and laboratory markers as well as for studies investigating the implications of vitamin D deficiency/supplementation on overall health/clinical outcomes of patients with/without COVID-19. 

## Figures and Tables

**Table 1:** Demographics and clinical characteristics of children.

Characteristics	COVID-19 78 (32.5)					NonCOVID-19 162 (67.5)					COVID-19 vs. Non-COVID-19
	Total COVID	Vitamin D				Total NonCOVID	Vitamin D				
		Def. 6 (7.69)	Insuf. 15 (19.23)	Normal 57 (73.08)	*p*		Def. 24 (14.81)	Insuf. 44 (27.16)	Normal 94 (58.02)	*p*	*p*
**Gender**											
Male	41 (52.56)	3 (50.00)	8 (53.33)	30 (52.63)	0.990	92 (56.79)	10 (41.67)	27 (61.36)	55 (58.51)	0.256	0.537
Female	37 (47.44)	3 (50.00)	7 (46.67)	27 (47.37)		70 (43.21)	14 (58.33)	17 (38.64)	39 (41.49)		
**Age**											
median (IQR)	10.00 (6; 27)	118.50 (46; 179)	21.00 (8; 52)	9.00 (6; 20)	0.010	90.50 (31; 135)	129.50 (93; 178)	119.00 (91; 157)	43.50 (17; 101)	0.000	0.000
<1 year	45 (57.69)	1 (16.67)	6 (40.00)	38 (66.67)	0.000	23 (14.20)	1 (4.17)	1 (2.27)	21 (22.34)	0.000	0.000
1–2 years	10 (12.82)	0 (0.00)	2 (13.33)	8 (14.04)		13 (8.02)	1 (4.17)	0 (0.00)	12 (12.77)		
2–6 years	15 (19.23)	1 (16.67)	4 (26.67)	10 (17.54)		32 (19.75)	0 (0.00)	5 (11.36)	27 (28.72)		
6–10 years	2 (2.56)	1 (16.67)	0 (0.00)	1 (1.75)		39 (24.07)	6 (25.00)	17 (38.64)	16 (17.02)		
>10 years	6 (7.69)	3 (50.00)	3 (20.00)	0 (0.00)		55 (33.95)	16 (66.67)	21 (47.73)	18 (19.15)		
**Symptoms**											
fever	68 (87.18)	4 (66.67)	14 (93.33)	50 (87.72)	0.249	11 (6.79)	2 (8.33)	2 (4.55)	7 (7.45)	0.777	0.000
loss of appetite	45 (57.69)	2 (33.33)	8 (53.33)	35 (61.40)	0.387	12 (7.41)	2 (8.33)	4 (9.09)	6 (6.38)	0.837	0.000
cough	37 (47.44)	2 (33.33)	4 (26.67)	31 (54.39)	0.124	7 (4.32)	1 (4.17)	1 (2.27)	5 (5.32)	0.714	0.000
diarrhea	23 (29.49)	0 (0.00)	3 (20.00)	20 (35.09)	0.134	5 (3.09)	0 (0.00)	2 (4.55)	3 (3.19)	0.582	0.000
vomiting	26 (33.33)	1 (16.67)	4 (26.67)	21 (36.84)	0.505	8 (4.94)	0 (0.00)	5 (11.36)	3 (3.19)	0.057	0.000
headache	14 (17.95)	3 (50.00)	4 (26.67)	7 (12.28)	0.045	17 (10.49)	5 (20.83)	5 (11.36)	7 (7.45)	0.158	0.107
rhinorrhea	24 (30.77)	2 (33.33)	3 (20.00)	19 (33.33)	0.603	7 (4.32)	1 (4.17)	2 (4.55)	4 (4.26)	0.996	0.000
nasal congest.	39 (50.00)	4 (66.67)	7 (46.67)	28 (49.12)	0.687	10 (6.17)	2 (8.33)	1 (2.27)	7 (7.45)	0.447	0.000
rash	19 (24.36)	0 (0.00)	1 (6.67)	18 (31.58)	0.048	3 (1.85)	0 (0.00)	1 (2.27)	2 (2.13)	0.765	0.000
breathing diff.	15 (19.23)	0 (0.00)	0 (0.00)	15 (26.32)	0.033	0 (0.00)	0 (0.00)	0 (0.00)	0 (0.00)	-	0.000

IQR—interquartile range (IQR: 25th percentile–75th percentile); 25(OH)D levels: Def.—deficiency: <20 ng/mL, Insuf.—insufficiency: 20–30 ng/mL, Normal: ≥30 ng/mL; nasal congest.—nasal congestion; breathing diff.—breathing difficulties.

**Table 2:** Correlation between vitamin D and laboratory characteristics of children.

Laboratory Characteristics	All COVID-19		COVID-19 78 (32.5)				All NonCOVID-19		NonCOVID-19 162 (67.5)			
			≤2 Years		>2 Years				≤2 Years		>2 Years	
	r	*p*	r	*p*	r	*p*	r	*p*	r	*p*	r	*p*
calcium	0.170	0.345	0.272	0.209	0.036	0.920	0.353 **	0.000	0.390 *	0.027	0.162	0.077
magnesium	0.470 *	0.036	0.444	0.111	−0.371	0.468	0.350 **	0.000	0.060	0.776	0.270 **	0.005
urea	−0.169	0.145	−0.015	0.912	−0.212	0.357	−0.083	0.317	0.333	0.073	0.137	0.143
creatinine	−0.137	0.235	0.311 *	0.021	−0.547 **	0.008	−0.446 **	0.000	0.046	0.812	−0.281 **	0.002
iron	0.011	0.926	0.101	0.496	−0.245	0.285	0.027	0.771	0.305	0.108	0.090	0.392
AST	0.247 *	0.041	0.053	0.716	0.055	0.818	0.466 **	0.000	0.185	0.327	0.322 **	0.001
ALT	0.145	0.208	0.071	0.605	−0.121	0.593	0.124	0.129	−0.055	0.765	0.008	0.928
CRP	0.139	0.226	0.097	0.482	0.239	0.272	−0.155	0.075	−0.044	0.816	−0.125	0.209
hemoglobin	−0.132	0.249	0.266	0.050	−0.244	0.261	−0.395 **	0.000	−0.003	0.988	−0.214 *	0.018
leukocyte	−0.089	0.441	−0.146	0.286	0.075	0.735	0.203 *	0.010	0.056	0.744	0.063	0.489
neutrophils	−0.063	0.587	0.089	0.522	0.341	0.111	−0.435 **	0.000	0.195	0.254	−0.205 *	0.023
lymphocytes	0.028	0.811	−0.078	0.576	−0.254	0.242	0.428 **	0.000	−0.154	0.371	0.215 *	0.017
monocytes	0.160	0.164	−0.055	0.695	−0.204	0.350	0.000	0.999	0.021	0.904	0.007	0.937
eosinophils	−0.023	0.842	0.032	0.817	−0.265	0.223	0.109	0.173	−0.043	0.804	0.054	0.555
basophils	0.109	0.344	0.148	0.279	−0.146	0.507	−0.113	0.156	0.029	0.867	−0.108	0.233
platelet	−0.080	0.485	−0.192	0.161	−0.094	0.668	0.079	0.322	0.000	0.998	−0.074	0.413
NLR	−0.040	0.731	0.095	0.494	0.292	0.176	−0.439 **	0.000	0.113	0.510	−0.209 *	0.020
PLR	−0.062	0.594	−0.043	0.755	0.227	0.297	−0.267 **	0.001	−0.014	0.938	−0.197 *	0.029
LMR	−0.123	0.287	−0.028	0.840	−0.253	0.244	0.332 **	0.000	−0.028	0.872	0.166	0.066
RBC	0.048	0.676	0.283 *	0.036	−0.078	0.723	−0.313 **	0.000	−0.213	0.213	−0.172	0.055
MCV	−0.330 *	0.003	−0.237	0.081	−0.367	0.085	−0.355 **	0.000	0.109	0.526	−0.277 **	0.002
MCH	−0.270 *	0.017	−0.210	0.123	0.014	0.948	−0.183 *	0.020	0.055	0.750	−0.063	0.484
MCHC	0.093	0.419	0.102	0.460	0.553 **	0.006	0.287 **	0.000	−0.069	0.690	0.339 **	0.000

AST—asparate aminotransferase, ALT—alanine aminotransferase, CRP—C-reactive protein, NLR—neutrophil lymphocyte ratio, PLR—platelet lymphocytes ratio, LMR—lymphocytes monocytes ratio, RBC—erythrocite, MCV—erythrocite mean cell volume, MCH—mean cell hemoglobin, MCHC—mean cell hemoglobin concentration, *, ** correlation is significant at 0.05, 0.01 level.

**Table 3:** Comparison of median values of laboratory characteristics according to vitamin D levels and age.

	≤2 Years				>2 Years					
	COVID-19, vit. D < 30	COVID-19, vit. D ≥ 30	NonCOVID-19, vit. D < 30	NonCOVID-19, vit. D ≥ 30	*p*	COVID-19, vit. D < 30	COVID-19, vit. D ≥ 30	NonCOVID-19, vit. D < 30	NonCOVID-19, vit. D ≥ 30	*p*
calcium	2.36 (2.28; 2.48)	2.40 (2.34; 2.48)	2.49 (2.41; 2.57)	2.53 (2.47; 2.60)	0.001	2.41 (2.27; 2.43)	2.45 (2.36; 2.47)	2.41 (2.35; 2.49)	2.43 (2.38; 2.51)	0.354
magnesium	0.83 (0.76; 0.89)	0.97 (0.92; 1.01)	0.86 (0.81; 0.90)	0.87 (0.85; 0.93)	0.032	0.82 (0.80; 0.88)	0.86 (0.86; 0.86)	0.81 (0.79; 0.85)	0.85 (0.80; 0.88)	0.023
urea	17.00 (14.00; 20.00)	18.00 (13.00; 22.00)	16.00 (7.00; 47.00)	18.00 (15.00; 22.00)	0.913	20.00 (17.50; 27.60)	19.00 (17,00; 27.00)	25.00 (21.00; 30.00)	27.00 (22.00; 31.00)	0.060
creatinine	0.39 (0.35; 0.41)	0.43 (0.37; 0.46)	0.36 (0.32; 0.44)	0.41 (0.38; 0.43)	0.197	0.60 (0.50; 0.72)	0.48 (0.45; 0.55)	0.57 (0.52; 0.65)	0.52 (0.47; 0.60)	0.011
iron	5.53 (3.61; 8.01)	6.68 (3.86; 9.06)	7.02 (6.05; 7.98)	11.56 (8.72; 14.93)	0.002	5.27 (2.48; 14.67)	4.25 (2.74; 7.48)	12.51 (9.30; 18.20)	14.40 (7.34; 19.21)	0.000
AST	53.00 (47.00; 71.00)	55.00 (41.50; 64.00)	37.50 (26.00; 49.00)	41.00 (36.50; 49.50)	0.021	27.00 (21.50; 48.00)	39.00 (30.00; 43.00)	23.00 (18.00; 29.00)	30.00 (24.50; 33.00)	0.001
ALT	27.00 (23.00; 41.00)	29.00 (20.00; 40.00)	22.00 (11.00; 49.00)	24.00 (17.00; 31.00)	0.335	18.50 (14.50; 26.00)	16.50 (12.00; 21.00)	16.00 (12.00; 22.00)	16.00 (13.00; 24.00)	0.830
CRP	3.51 (2.00; 6.97)	4.35 (2.04; 11.86)	2.00 (2.00; 2.00)	2.00 (2.00; 2.00)	0.000	7.69 (2.00; 25.41)	9.46 (2.00; 26.50)	2.00 (2.00; 3.00)	2.00 (2.00; 2.00)	0.009
hemoglobin	9.60 (9.40; 10.40)	11.10 (10.20; 11.70)	10.30 (7.20; 11.10)	11.40 (10.80; 11.80)	0.004	12.80 (11.85; 13.40)	12.60 (11.80; 13.40)	13.40 (12.40; 14.20)	12.58 (11.90; 13.60)	0.039
leukocyte	6.98 (4.82; 7.91)	6.31 (4.69; 9.94)	9.76 (8.34; 12.32)	9.67 (7.91; 12.10)	0.006	6.99 (5.10; 9.59)	6.95 (5.49; 8.71)	7.28 (6.28; 9.11)	7.35 (6.31; 8.95)	0.741
neutrophils	37.10 (31.30; 47.30)	41.10 (24.90; 53.80)	18.60 (11.50; 29.20)	16.70 (13.80; 23.60)	0.000	64.60 (47.20; 76.50)	69.20 (52.10; 84.00)	48.80 (37.40; 53.40)	40.90 (29.70; 53.30)	0.000
lymphocytes	44.90 (29.70; 57.60)	42.60 (31.30; 56.10)	72.60 (55.60; 79.60)	70.50 (61.50; 74.80)	0.000	25.15 (16.15; 41.65)	24.40 (11.30; 33.60)	40.00 (34.50; 50.10)	46.10 (34.75; 54.70)	0.000
monocytes	15.60 (12.70; 19.60)	15.20 (9.90; 21.00)	6.50 (6.10; 12.30)	9.20 (7.50; 10.40)	0.000	7.50 (5.75; 10.80)	5.50 (4.40; 8.60)	8.70 (7.20; 10.10)	9.00 (7.60; 10.70)	0.020
eosinophils	0.20 (0.00; 0.80)	0.35 (0.00; 1.10)	2.40 (2.30; 2.50)	2.80 (2.40; 3.50)	0.000	0.15 (0.00; 3.10)	0.30 (0.00; 0.70)	2.40 (1.40; 4.60)	2.70 (1.60; 3.90)	0.000
basophils	0.30 (0.20; 0.40)	0.20 (0.20; 0.50)	0.20 (0.10; 0.30)	0.30 (0.20; 0.40)	0.669	0.15 (0.10; 0.45)	0.20 (0.10; 0.30)	0.30 (0.20; 0.50)	0.30 (0.20; 0.50)	0.010
platelet	348.00 (315.00; 422.00)	283.50 (230.00; 368.00)	356.00 (251.00; 644.00)	360.00 (291.00; 446.00)	0.038	240.50 (220.00; 316.50)	263.00 (212.00; 324.00)	315.00 (245.00; 372.00)	281.50 (223.00; 345.00)	0.201
NLR	0.90 (0.64; 1.44)	0.99 (0.46; 0.68)	0.26 (0.14; 0.53)	0.24 (0.19; 0.40)	0.000	2.57 (1.23; 4.64)	2.84 (1.55; 7.43)	0.24 (0.80; 1.59)	0.88 (0.52; 1.60)	0.000
PLR	7.85 (6.31; 12.80)	7.30 (5.32; 11.19)	4.90 (3.15; 11.58)	5.39 (4.17; 6.68)	0.012	12.97 (5.54; 15.48)	10.78 (6.42; 30.97)	8.20 (5.88; 10.65)	6.05 (4.89; 9.01)	0.003
LMR	1.94 (1.54; 4.95)	2.58 (1.81; 4.52)	11.90 (4.52; 12.25)	7.69 (5.88; 9.72)	0.000	4.70 (1.83; 5.80)	3.36 (2.46; 4.44)	4.47 (3.69; 5.72)	5.04 (3.91; 6.24)	0.038
RBC	4.02 (3.32; 4.40)	4.27 (3.93; 4.59)	4.99 (4.27; 5.20)	4.24 (4.10; 4.52)	0.090	4.44 (4.29; 4.54)	4.40 (4.20; 4.71)	4.73 (4.43; 5.01)	4.65 (4.31; 4.87)	0.039
MCV	77.30 (64.50; 84.30)	77.80 (73.80; 80.80)	70.00 (65.60; 70.50)	76.40 (74.30; 79.00)	0.191	82.45 (78.90; 83.10)	79.10 (76.90; 81.50)	82.30 (80.10; 85.40)	79.20 (76.90; 83.20)	0.001
MCH	27.00 (21.80; 28.90)	27.00 (25.60; 28.00)	22.20 (20.40; 24.10)	26.70 (26.20; 27.80)	0.170	28.10 (27.55; 28.90)	28.10 (27.10; 28.90)	28.20 (27.20; 28.90)	27.80 (26.80; 29.00)	0.639
MCHC	34.30 (33.80; 35.00)	34.60 (34.10; 35.10)	34.10 (31.50; 34.40)	34.80 (34.30; 35.20)	0.123	34.60 (34.40; 35.30)	35.40 (35.10; 36.00)	34.00 (33.20; 34.90)	35.00 (34.20; 35.50)	0.000

AST—asparate aminotransferase, ALT—alanine aminotransferase, CRP—C-reactive protein, NLR—neutrophil lymphocyte ratio, PLR—platelet lymphocytes ratio, LMR—lymphocytes monocytes ratio, RBC—erythrocite, MCV—erythrocite mean cell volume, MCH—mean cell hemoglobin, MCHC—mean cell hemoglobin concentration.

## Data Availability

The data presented in this study are available on reasonable request from the corresponding author.
